# Distinct Mechanisms Account for In Vitro Activation and Sensitization of TRPV1 by the Porphyrin Hemin

**DOI:** 10.3390/ijms221910856

**Published:** 2021-10-08

**Authors:** Natalie E. Palmaers, Steffen B. Wiegand, Christine Herzog, Frank G. Echtermeyer, Mirjam J. Eberhardt, Andreas Leffler

**Affiliations:** Department of Anesthesiology and Intensive Care Medicine, Hannover Medical School, 30625 Hannover, Germany; Natalie.Palmaers@stud.mh-hannover.de (N.E.P.); wiegand.steffen@googlemail.com (S.B.W.); herzog.christine@mh-hannover.de (C.H.); echtermeyer.frank@mh-hannover.de (F.G.E.); eberhardt.mirjam@mh-hannover.de (M.J.E.)

**Keywords:** sensory neuron, nociceptor, TRPV1, calcium influx, photosensitization, pain, oxidative stress

## Abstract

TRPV1 mediates pain occurring during sickling episodes in sickle cell disease (SCD). We examined if hemin, a porphyrin released during intravascular hemolysis modulates TRPV1. Calcium imaging and patch clamp were employed to examine effects of hemin on mouse dorsal root ganglion (DRG) neurons and HEK293t cells expressing TRPV1 and TRPA1. Hemin induced a concentration-dependent calcium influx in DRG neurons which was abolished by the unspecific TRP-channel inhibitor ruthenium red. The selective TRPV1-inhibitor BCTC or genetic deletion of TRPV1 only marginally impaired hemin-induced calcium influx in DRG neurons. While hTRPV1 expressed in HEK293 cells mediated a hemin-induced calcium influx which was blocked by BCTC, patch clamp recordings only showed potentiated proton- and heat-evoked currents. This effect was abolished by the PKC-inhibitor chelerythrine chloride and in protein kinase C (PKC)-insensitive TRPV1-mutants. Hemin-induced calcium influx through TRPV1 was only partly PKC-sensitive, but it was abolished by the reducing agent dithiothreitol (DTT). In contrast, hemin-induced potentiation of inward currents was not reduced by DTT. Hemin also induced a redox-dependent calcium influx, but not inward currents on hTRPA1. Our data suggest that hemin induces a PKC-mediated sensitization of TRPV1. However, it also acts as a photosensitizer when exposed to UVA-light used for calcium imaging. The resulting activation of redox-sensitive ion channels such as TRPV1 and TRPA1 may be an in vitro artifact with limited physiological relevance.

## 1. Introduction

The ability of sensory neurons to detect painful noxae largely depends on irritant receptors such as the capsaicin receptor TRPV1 (transient receptor potential cation channel subfamily V member 1) which is gated by several exogenous and endogenous substances [1]. TRPV1-expressing sensory neurons are regarded to be mainly nociceptors, e.g., predominantly afferent C-fibers responsible for peripheral pain signaling [2,3]. In addition to mediating pain, TRPV1-expressing sensory neurons also seem to maintain organ function and to fulfil protective effects in several disorders [4,5,6,7,8,9,10]. A recent study from Xu and colleagues describes that TRPV1-expressing sensory neurons have an important protective role in the hematologic disorder sickle cell disease (SCD) [4]. In a mouse model of SCD, the ablation of TRPV1-expressing sensory neurons resulted in an aggravated phenotype with more severe vaso-occlusive episodes, resulting in organ dysfunction and an increased lethality. On the other hand, TRPV1 was shown to be responsible for severe pain symptoms occurring during sickling episodes in SCD mice [11,12]. In subsequent studies, different mechanisms were identified which seem to trigger this TRPV1-dependent pain phenotype. Vincent and colleagues found that pain behavior in SCD mice critically depends on an activation of mast cells [13]. Among several proalgesic cytokines which are released from mast cells, tryptase may sensitize TRPV1 by activating the PAR2 receptor expressed in sensory neurons. More recently, Sadler et al. found that increased levels of chemokine ligand 2 lead to a sensitization of TRPV1 via the chemokine receptor 2 [14]. Consequently, systemic inhibition of chemokine receptor 2 alleviated pain symptoms in SCD mice. Considering the known polymodality of TRPV1, it is not surprising that several signaling pathways being activated in the course of SCD can trigger pain by mediating an activation or sensitization of TRPV1. In the present study, we explored the effects of the porphyrin hemin (or heme) on TRPV1. Hemin is the prosthetic group of hemoproteins crucial for oxygen binding and transport [15]. However, when excessively released in pathophysiological states such as hemolysis, or in the course of SCD or blood transfusions, unbound “free hemin” is toxic, and seems to aggravate organ dysfunction by inducing oxidative stress, inflammation, and cytotoxicity [15]. Accordingly, free hemin impacts the severity of SCD [16,17]. While the effects of hemin on sensory neurons have not yet been explored, it induces a concentration-dependent calcium influx in cortical neurons [18]. Hemin is also a potent modulator of voltage-gated potassium channels [19,20,21], and it was reported to activate PKC and to induce oxidative stress [15,22]. Indeed, PKC is known to sensitize TRPV1 and to trigger TRPV1-dependent hyperalgesia [23,24]. Furthermore, TRPV1 is gated by oxidation [25]. Given these properties of hemin together with the strong evidence for a prominent role of TRPV1 in SCD, we hypothesized that hemin may sensitize or even activate TRPV1. We employed in vitro electrophysiology and calcium imaging on DRG neurons as well as on recombinant TRPV1 channels. Our data indicate that hemin might be a relevant endogenous modulator of TRPV1.

## 2. Results

### 2.1. Hemin Induces a Calcium Influx in Mouse DRG Neurons

**Figure 1:**
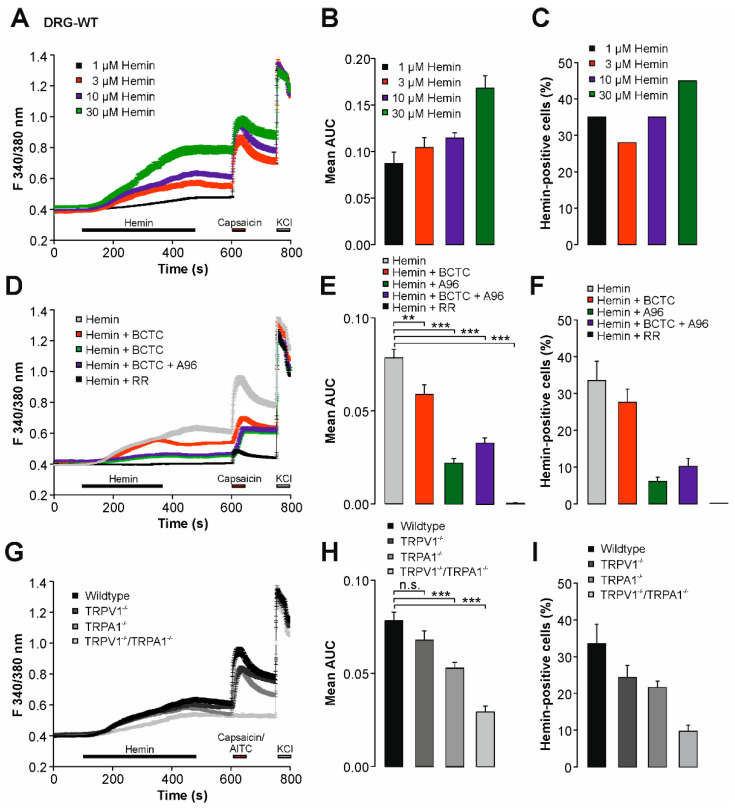
Hemin induces an increase in intracellular calcium in DRG neurons. (**A**) Concentration-dependent increase in intracellular calcium by hemin in wildtype DRG neurons. Hemin at 1, 3, 10, and 30 µM was applied for 300 s followed by 1 µM capsaicin for verification of TRPV1 expression and 40 mM KCl for identification of excitable cells. (**B**) Mean area under the curve (AUC) for hemin-induced calcium responses at different concentrations. (**C**) Mean percentages of hemin-sensitive DRG neurons at 1, 3, 10, and 30 µM hemin. (**D**) Mean calcium influx on wildtype DRG neurons induced by 1 µM hemin applied alone or in combination with BCTC, A967079, BCTC + A967079, or ruthenium red. Note that the combinations of hemin with inhibitors was only applied for 240 s, instead of 300 s for hemin applied alone (**E**) Mean area under the curve (AUC) for hemin-induced calcium responses in wildtype DRG neurons in presence of different inhibitors. The AUCs were calculated for 240 s application. (**F**) Mean percentages of hemin-sensitive DRG neurons at 10 µM hemin applied alone or combination with different blockers. (**G**) Mean calcium influx induced by 10 µM hemin in wildtype, TRPV1-knockout, TRPA1-knockout, or TRPV1/TRPA1-knockout DRG neurons. (**H**) Mean area under the curve (AUC) for hemin-induced calcium responses in DRG neurons of each genotype. (**I**) Mean percentages of hemin-sensitive DRG neurons derived from different genotypes, e.g., wildtype, TRPV1-knockout, TRPA1-knockout, and TRPV1/TRPA1-knockout. The AUCs were calculated for 240 s application. All data are shown as mean ± S.E.M. ** denotes *p* < 0.01, *** denotes *p* < 0.001.

Hemin was previously demonstrated to induce an acute increase in intracellular calcium in cortical neurons [18]. We started this study by exploring the effects of hemin on mouse DRG neurons by means of ratiometric calcium imaging recordings. As is demonstrated in Figure 1A,B, application of hemin at 1 to 30 µM provoked a calcium influx with concentration-dependent magnitudes (ANOVA F(3, 1550) = 8.649, *p* < 0.001, followed by HSD posthoc test). The percentage of hemin-sensitive cells only slightly increased with higher concentrations of hemin (Figure 1C, 1 µM: 35%, 3 µM: 28%, 10 µM: 35%, and 30 µM: 45%). In order to examine if TRPV1 is relevant for this hemin sensitivity, we next co-applied hemin with the unselective TRP-channel blocker ruthenium red (RR, 10 µM) or the TRPV1-selective inhibitor BCTC (100 nM)). As is demonstrated in Figure 1D,E, 10 µM RR abolished calcium influx induced by 10 µM hemin (n = 635, *p* < 0.001). Indeed, 0% of the cells displayed hemin sensitivity in presence of RR. In contrast, BCTC (Figure 1F, n = 411, *p* = 0.004) only marginally reduced the magnitude of hemin-induced calcium influx (Figure 1D,E, ANOVA F(4, 3003)=80.369, *p* < 0.001, HSD post hoc test, *p*-values are displayed in comparison to cells treated with 10 µM hemin alone). Accordingly, the percentage of hemin-sensitive neurons was only marginally reduced by BCTC as well (Figure 1F, from 34 ± 5% to 28 ± 4%). This striking difference between RR and BCTC indicated the involvement of another TRP-channel or alternative mechanisms. We hypothesized that the polymodal irritant receptor TRPA1 might be involved, and therefore examined the effect of the TRPA1-selective inhibitor A967079. Indeed, inhibition of TRPA1 reduced both the magnitude (Figure 1D,E), n = 343, *p* < 0.001) of hemin-induced responses and the percentage (Figure 1F, 6 ± 2%) of hemin-sensitive DRG neurons. When BCTC was applied together with A967079 (n = 708, *p* < 0.001), the magnitude of hemin-induced calcium influx and the percentage of hemin-sensitive neurons (10 ± 2%) did not further decrease (Figure 1E,F). These data indicate a prominent role for TRPA1, but not for TRPV1 in regard to hemin-induced calcium influx. We next examined DRG neurons derived from mice lacking TRPV1, TRPA1, or both TRPV1 and TRPA1. As is demonstrated in Figure 1G,H, the deletion of only TRPV1 (n = 475, *p* = 0.382) had a small effect on the magnitude of hemin-induced calcium influx as compared to effects observed in DRG neurons from wildtype mice (n = 911). However, the fraction of hemin-sensitive cells dropped to 24 ± 3% in TRPV1-null DRG neurons (Figure 1I). The deletion of TRPA1 produced similar effects, e.g., a small reduction in the magnitude of hemin influx effects (n = 762, *p* < 0.001) as well as a reduction in responsive cells to 22 ± 2% (Figure 1G,H). In perfect agreement with the pharmacological experiments, the reduction in hemin sensitivity was rather prominent in TRPV1/TRPA1 double-knockout neurons, both in regard to magnitude (n = 405, *p* < 0.001) and the fraction of hemin-sensitive cells (Figure 1F, 10 ± 2%). Taken together, these data suggest that both TRPV1 and TRPA1 seem to be relevant to hemin-induced increase in intracellular calcium in DRG neurons (ANOVA F(3, 2549) = 19.632, *p* < 0.001, HSD post hoc test; if not mentioned otherwise *p*-values are displayed in comparison to wildtype).

### 2.2. Hemin Induces an Activation and Sensitization of hTRPV1 Expressed in HEK293t Cells

**Figure 2:**
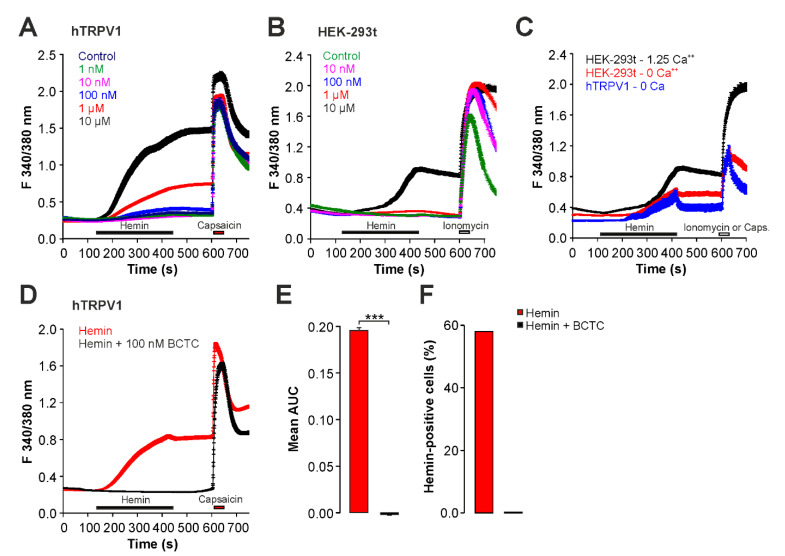
Hemin activates hTRPV1 when examined with calcium imaging. (**A**,**B**) Concentration-dependent activation of increase in intracellular calcium by hemin in HEK293t cells expressing hTRPV1 (**A**) or untransfected HEK293t cells (**B**). Hemin was applied for 300 s followed by washout and capsaicin (100 nM) or ionomycin (5 µM). Note that 1 µM hemin induces calcium responses only in cells expressing hTRPV1. (**C**) Hemin-induced (10 µM) calcium responses in naïve HEK-293t cells (red) or hTRPV1-expressing cells (blue) in nominal calcium-free extracellular solution. Following washout, capsaicin or ionomycin were applied in calcium-containing solution. For comparison, the calcium response of HEK293t cells to 10 µM hemin in calcium-containing solution is also displayed (black). (**D**) Hemin-induced (1 µM) calcium responses in hTRPV1-expressing cells with or without (application of) the TRPV1-inhibitor BCTC (100 nM). Following washout, capsaicin (100 nM) was applied to verify expression of hTRPV1. (**E**) Area under the curve (AUC) for hemin-induced increase in fluorescence ratio described in (**D**). ANOVA F(5, 3787) = 196.42, *p* < 0.001. (**F**) Fraction of hTRPV1-expressing cells displaying an increased intracellular calcium following hemin or hemin in combination with BCTC. *** denotes *p* < 0.001.

As this study primarily aimed to describe hemin-induced effects on TRPV1, we next employed HEK293t cells expressing recombinant hTRPV1, and explored these by means of calcium imaging and whole-cell patch clamp recordings. Similar to the effects observed in DRG neurons, HEK293t cells expressing hTRPV1 exhibited an increase in cytosolic calcium when exposed to hemin (Figure 2A, n > 400 for each concentration). Surprisingly, hemin also induced calcium influx in untransfected HEK293t cells (Figure 2B, n > 200 for each concentration). Thus, similar to DRG neurons, hemin obviously triggers a TRPV1/TRPA1-independent calcium influx in HEK293t cells. Although not a primary endpoint in this study, we asked this TRPV1/TRPA-independent response in naïve HEK293t is due to a calcium influx or rather to a release of calcium from intracellular stores. As is demonstrated in Figure 2C, calcium imaging experiments performed in nominal calcium-free solution (0 calcium, 5 mM EGTA, n = 300) revealed a small increase in intracellular calcium when 10 µM hemin was applied. However, the effect was considerably smaller than the effect observed in presence of extracellular calcium. When the same experiments were performed with HEK293 cells expressing hTRPV1 (n = 65), an almost identical effect was observed (Figure 2C). These data indicate that when hemin is investigated by means of calcium imaging, the resulting increase in intracellular calcium is due to both a calcium influx and release of calcium from internal stores. Nonetheless, cells expressing hTRPV1 seem to be more sensitive to hemin as compared to naïve HEK293t cells. Indeed, inhibition of hTRPV1 by BCTC completely blocked calcium influx induced by 1 µM hemin in cells expressing hTRPV1 (Figure 2D,E, n = 540). Accordingly, the percentage of hemin-sensitive cells dropped from 58% to 0.2% when BCTC was co-applied with hemin (Figure 2F).

**Figure 3:**
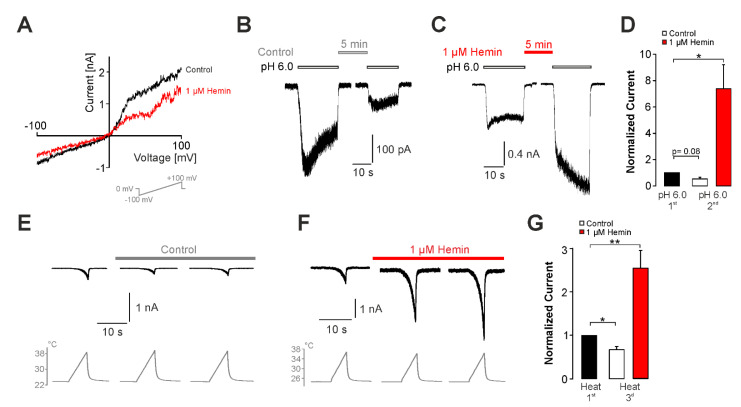
Hemin sensitizes hTRPV1 when examined with patch clamp electrophysiology. (**A**) Representative whole-cell patch clamp recording on a HEK293t cell expressing hTRPV1. Membrane currents were evoked by 500 ms long voltage-ramps from -100 to +100 mV. Note that hemin reduces the membrane current. (**B**,**C**) Whole-cell patch clamp recordings on hTRPV1-expressing cells challenged with two consecutive applications of pH 6.0 with control solution (B or 1 µM hemin (**C**) applied for 5 min between applications of acidic solution. (**D**) Mean normalized peak amplitudes of inward currents evoked by pH 6.0 in (**B**,**C**). Current amplitudes were normalized to the amplitude of the first current. (**E**,**F**) Typical heat-evoked inward currents in cells expressing hTRPV1. Following the first challenge with heat, cells were treated either with control solution (**E**) or hemin (**F**) and the depicted currents were recorded after 3 or 6 min, respectively. (**G**) Mean normalized peak amplitudes of inward currents evoked by heat in (**E**,**F**). Current amplitudes recorded after 6 min were normalized to the amplitude of the first current. Cells were held at -60 mV in all patch clamp experiments. Data are shown as mean ± S.E.M. * denotes *p* < 0.05, ** denotes *p* < 0.01.

When the same concentration of hemin (1 µM) was applied on hTRPV1-HEK293t cells in whole-cell patch clamp experiments, however, we observed an hemin-induced reduction in the basal leak current, rather than an activation, even when monitored for several minutes (Figure 3A, n = 8). Application of 10 µM hemin also did not result in an activation of hTRPV1, but rather in a rapid loss of the seal formation (data not shown). In order to examine if hemin might sensitize rather than directly activate hTRPV1, the effects of hemin on proton and heat-evoked currents were examined. When hTRPV1 was repeatedly activated by protons (pH 6.0), the current resulting from the second challenge with pH 6.0 displayed a non-significant tachyphylaxis when control solution was applied during the 5 min long washout of the acidic solution (Figure 3B, n = 11, paired *t*-test, *p* = 0.083). When 1 µM hemin was applied for 5 min, however, the second proton-evoked inward currents displayed a significant increase (Figure 3C,D, n = 11, paired *t*-test, *p* < 0.05). A similar effect was observed on heat-evoked currents, e.g., when hTRPV1 was activated by three consecutive heat-stimuli, inward currents displayed a significant tachyphylaxis when control solution was applied (Figure 3E, n = 11, paired *t*-test, *p* < 0.05). When 1 µM hemin was applied between the applications of heated solution, hTRPV1 generated significantly larger inward currents as compared to the initial heat-evoked current (Figure 3F,G, n = 11, paired *t*-test, *p* < 0.01).

**Figure 4:**
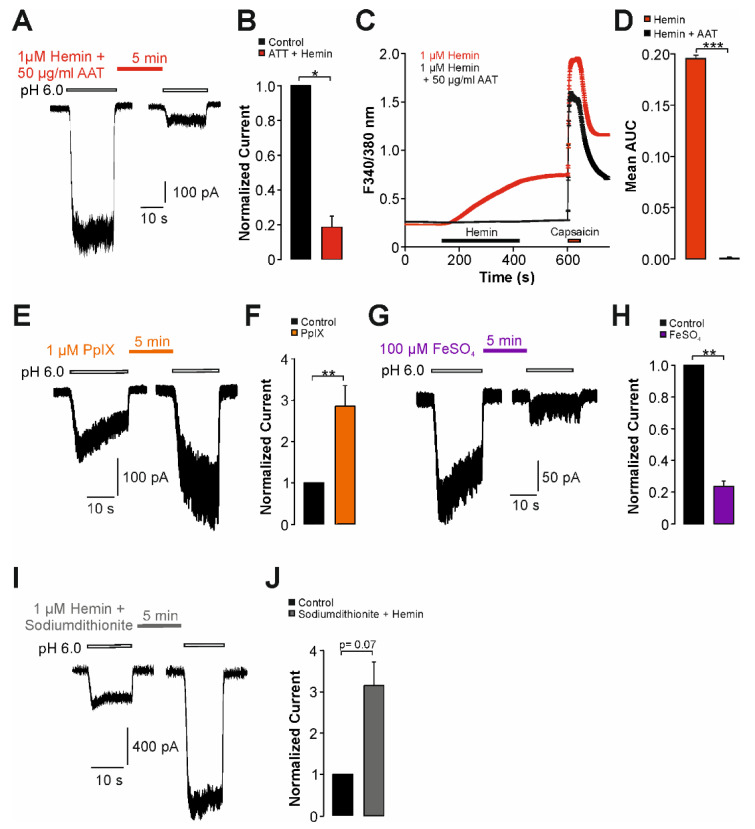
Determinants of hemin-induced activation of hTRPV1. (**A**) Patch clamp recordings on hTRPV1-expressing cells challenged with two consecutive applications of pH 6.0 with 1 µM hemin and 50 µg/mL α1-antitrypsin (AAT) applied for 5 min between applications of protons. (**B**) Mean normalized peak amplitudes of inward currents evoked by pH 6.0 in (**A**). Current amplitudes were normalized to the first current. (**C**) Hemin-induced (1 µM) calcium responses in hTRPV1-expressing cells applied with or without application of 50 µg/ml AAT. (**D**) Area under the curve (AUC) for hemin-induced increase in fluorescence ratio described in (**C**). ANOVA F(5,3787) = 196.42, *p* < 0.001. (**E,G,I**) Patch clamp recordings on hTRPV1-expressing cells challenged with two consecutive applications of pH 6.0 with 1 µM PpIX (**E**), 100 µM FeSO4 (**G**), or hemin and 10 µM sodiumdithionite (**I**) applied for 5 min between applications of protons. (**F,H,J**) Mean normalized peak amplitudes of inward currents evoked by pH 6.0 in (**E,G,I**), respectively. Current amplitudes were normalized to the first current. Cells were held at −60 mV in all patch clamp experiments. Data are shown as mean ± S.E.M. * denotes *p* < 0.05, ** denotes *p* < 0.01, *** denotes *p* < 0.001.

We next aimed to ascertain which properties of hemin are mediating this sensitization of TRPV1. Human α1-antitrypsin was previously demonstrated to act as a scavenger of hemin and to prevent hemin-induced effects [26]. When hemin was co-applied with α1-antitrypsin (50 µg/mL), proton-evoked currents generated by hTRPV1 displayed a tachyphylaxis instead of a potentiation (Figure 4A,B, n = 11, paired *t*-test, *p* < 0.05). Furthermore, α1-antitrypsin more or less completely prevented hemin-induced calcium influx in HEK293t cells expressing hTRPV1 (Figure 4C,D, n = 1195). Indeed, only 0.4% of capsaicin-sensitive cells responded to hemin when α1-antitrypsin was co-applied. Hemin is a complex of protoporphyrin IX (PpIX) and iron. We examined if application of any of these two substances alone sensitizes hTRPV1. As is demonstrated in Figure 4E,F, 1 µM PpIX induced a significant increase in acid-evoked inward currents (n = 13, paired *t*-test, *p* < 0.01). In contrast, application of 100 µM FeSO4 resulted in a strong tachyphylaxis (Figure 4G,H, n = 10, paired *t*-test, *p* < 0.01). Considering that hemin is the ferric state of free heme, which is rapidly oxidized when it is released from cells, we asked if heme itself is sensitizing hTRPV1 to a similar extent as observed for hemin. In order to obtain free heme, hemin was incubated with the reducing agent sodiumdithionite (10 µM). Although not statistically significant, the mixture of hemin and sodiumdithionite seemed to induce a sensitization of hTRPV1 for activation by pH 6.0 (Figure 4I,J, n = 9, paired *t*-test, *p* = 0.067) Taken together, these data suggest that unbound hemin sensitizes hTRPV1 by a mechanism requiring the porphyrin molecule rather than iron.

### 2.3. Sensitization of TRPV1 by Hemin Is Mediated by PKC

**Figure 5:**
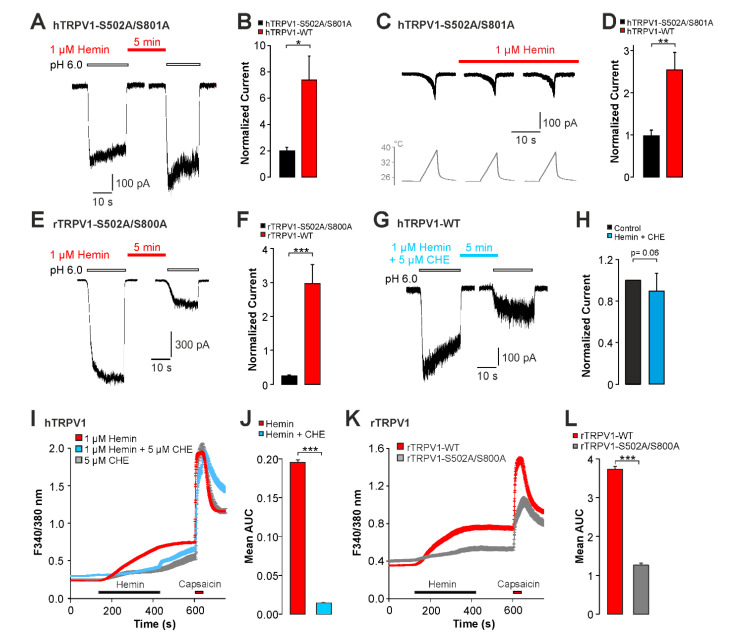
Hemin sensitizes TRPV1 by a PKC-dependent mechanism. (**A**) Whole-cell patch clamp recordings on cells expressing hTRPV1-S502A/S801A challenged with pH 6.0 with 1 µM hemin applied for 5 min between applications of protons. (**B**) Mean normalized peak amplitudes of inward currents evoked by pH 6.0 in (**A**). For comparison, the data obtained on hTRPV1-WT was depicted. Current amplitudes were normalized to the amplitude of the first current. (**C**) Heat-evoked inward currents in cells expressing hTRPV1-S502A/S801A. Following the first challenge with heat, cells were treated with 1 µM hemin and the depicted currents were recorded after 3 and 6 min, respectively. (**D**) Mean normalized peak amplitudes of inward currents evoked by heat in (**C**) and for hTRPV1-WT. Current amplitudes recorded after 6 min were normalized to the amplitude of the first current. (**E**) Patch clamp recordings on rTRPV1-S502A/S800A-expressing cells challenged with two consecutive applications of pH 6.0 with 1 µM hemin applied for 5 min between applications of protons. (**F**) Mean normalized peak amplitudes of inward currents evoked by pH 6.0 in (**F**) and for rTRPV1-WT. Current amplitudes were normalized to the first current. (**G**) Patch clamp recordings on hTRPV1-WT-expressing cells challenged with two consecutive applications of pH 6.0 with 1 µM hemin and 5 µM CHE applied for 5 min between applications of protons. (**H**) Mean normalized peak amplitudes of inward currents evoked by pH 6.0 in (**G**). Current amplitudes were normalized to the first current. Cells were held at −60 mV in all patch clamp experiments. (**I**) Hemin-induced (1 µM) increase in intracellular calcium in hTRPV1-expressing cells with or without the PKC-inhibitor CHE (5 µM), or CHE applied alone. Following washout, capsaicin (1 µM) was applied for verify expression of hTRPV1. (**J**) Mean area under the curve (AUC) for hemin-induced increase in ratio described in (ANOVA F(5,3787) = 196.42, *p* < 0.001). (**K**) Hemin-induced (1 µM) calcium-increase in cells expressing rTRPV1-WT or rTRPV1-S502A/S800A. Following washout, capsaicin (1 µM) was applied to verify expression of rTRPV1. (**L**) Mean area under the curve (AUC) for hemin-induced increase in fluorescence ratio described in (unpaired *t*-test, n > 170 per group, *p* < 0.001). Data are shown as mean ± S.E.M. * denotes *p* < 0.05, ** denotes *p* < 0.01, *** denotes *p* < 0.001.

We then aimed to identify mechanisms mediating hemin-induced sensitization of hTRPV1. Among several mechanisms which have been described to modify the functional properties of TRPV1, we considered the involvement of proteinkinase C (PKC) as a likely candidate mechanism. Rodent orthologues of TRPV1 are known to be sensitized and even gated following activation of PKC [24], and the PKC-phosphorylation sites on TRPV1 are well defined [23]. Furthermore, hemin was demonstrated to activate PKC [22,26]. In order to examine the role of PKC for hemin-induced sensitization of TRPV1, we employed PKC-insensitive mutant constructs from both human (hTRPV1-S502A/S801A) and rat (rTRPV1- S502A/S800A) TRPV1. When compared to wildtype hTRPV1, hemin-induced potentiation of proton-evoked inward currents was diminished on hTRPV1-S502A/S801A (Figure 5A,B, n = 18, paired *t*-test, *p* = 0.33). Accordingly, hTRPV1-S502A/S801A was significantly less sensitized by hemin as compared to hTRPV1-WT (Figure 5B, unpaired *t*-test, *p* < 0.05) The corresponding experiment on heat-evoked currents gave the same result, i.e., hemin-induced potentiation of heat-induced currents on hTRPV1-S502A/S801A was reduced (Figure 5C,D, n = 8, unpaired *t*-test, *p* < 0.01). In order to corroborate this finding on human TRPV1, we also investigated the effects on hemin on rTRPV1 wildtype and the mutant rTRPV1-S502A/S800. Similar to hTRPV1, wildtype rTRPV1 (Figure 5F, n = 12, paired *t*-test, *p* < 0.05) displayed increased proton-evoked currents following treatment with 1 µM hemin. In contrast to rTRPV1-WT, rTRPV1-S502A/S800A displayed tachyphylaxis instead of sensitization (Figure 5E,F, n = 11, paired *t-*test, *p* < 0.01). Accordingly, the difference between wildtype and mutant rTRPV1 was highly significant (unpaired *t*-test, *p* < 0.001). We also asked if inhibition of PKC inhibits hemin-induced sensitization of TRPV1. As is demonstrated in Figure 5G,H, the PKC-inhibitor chelerythrine chloride completely inhibited hemin-induced sensitization of hTRPV1 (n = 12, paired *t*-test, *p* = 0.06). When examined by means of calcium imaging, chelerythrine chloride only partly inhibited hemin-induced calcium influx on hTRPV1 (Figure 5I,J, n = 328). Furthermore, 10% of capsaicin-sensitive cells remained hemin-sensitive in presence of chelerythrine chloride. Of note, application of 5 µm chelerythrine chloride alone also induced an increase in intracellular calcium which was only marginally smaller than the effect evoked by hemin and chelerythrine chloride applied together (Figure 5I, n = 237). Thus, the hemin-induced effect on hTRPV1 seems to be effectively blocked by chelerythrine chloride. Finally, hemin-induced calcium influx was reduced but still evident on rTRPV1-S502A/S800A when compared with rTRPV1 wildtype (Figure 5K,L, n = 171 and 405, respectively). This was also evident on the percentages of hemin-sensitive neurons; rTRPV1-S502A/S800A: 44 ± 8% versus rTRPV1-wildtype: 70 ± 6% (*p* = 0.015, n = 7–10, Mann–Whitney U-test). Thus, independent of species, PKC seems to be required for hemin-induced sensitization of TRPV1 observed in patch clamp experiments. For calcium imaging however, role of PKC for hemin-induced activation of TRPV1 is not quite clear.

### 2.4. UVA-Light Triggers Direct Activation of TRPV1 and TRPA1 by Hemin

**Figure 6:**
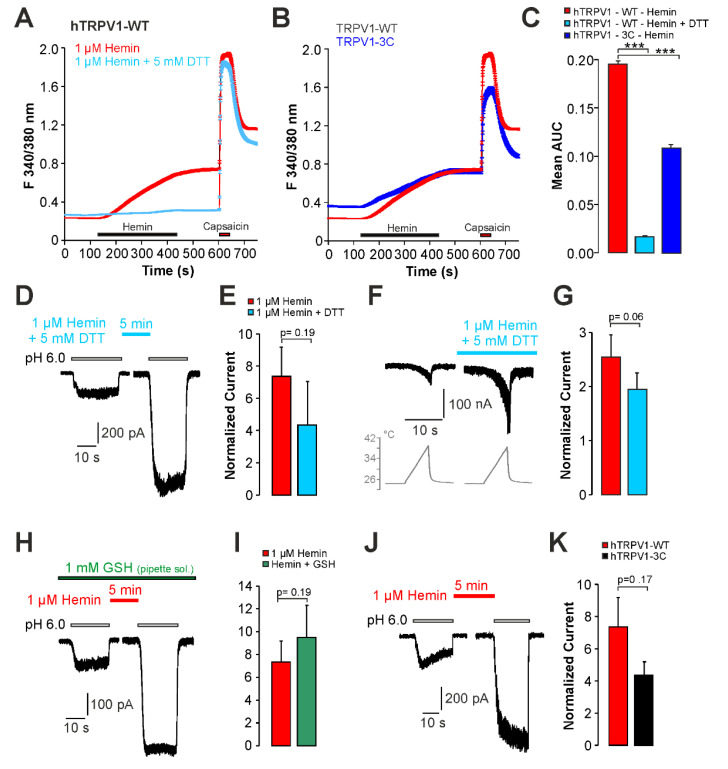
Hemin-induced increase in intracellular calcium is redox-dependent. (**A**) Mean calcium responses in cells expressing hTRPV1-WT treated with 1 µM hemin or hemin together with 5 mM DTT. (**B**) Mean calcium responses in cells expressing hTRPV1-WT or hTRPV1-3C treated with 1 µM hemin. In (**A**,**B**), capsaicin (1 µM) was applied to verify expression of hTRPV1. (**C**) Area under the curve (AUC) for hemin-induced increase in fluorescence ratio described in (**C,B**). ANOVA F(2, 2117)=88.167, *p* < 0.001. (**D**) Patch clamp recordings on hTRPV1-WT-expressing cells challenged with two consecutive applications of pH 6.0 with 1 µM hemin and 5 mM DTT applied for 5 min between applications of protons. (**E**) Mean peak amplitudes of inward currents evoked by pH 6.0 in (**C**) as well as of cells treated with hemin alone. The bars display normalized amplitudes of the currents resulting from the second application of pH 6.0. (**F**) Heat-evoked inward currents in cells expressing hTRPV1-WT. Following the first heat-evoked current, cells were treated with 1 µM hemin in combination with 5 mM DTT. The depicted current was recorded after 6 min. (**G**) Mean normalized peak amplitudes of inward currents evoked by heat in (**E**) and for cells treated with hemin only. (**H**) Proton-evoked inward currents on hTRPV1-WT treated with 1 µM hemin together with 1 mM GSH (in the patch pipette). (**I**) Mean peak amplitudes of inward currents evoked by pH 6.0 in (**G**) as well as of cells treated with hemin. The bars display normalized amplitudes of the currents resulting from the second application of pH 6.0. (**J**) Representative trace of proton-evoked inward current on hTRPV1-3C treated with 1 µM hemin for 5 min between two applications of pH 6.0. (**K**) Mean peak amplitudes of inward currents evoked by pH 6.0 in (**I**) as well as of cells expressing hTRPV1-WT. The bars display normalized amplitudes of the currents resulting from the second application of pH 6.0. Cells were held at −60 mV in all patch clamp experiments. Data are shown as mean ± S.E.M. *** denotes *p* < 0.001.

The discrepancy observed between patch clamp (no direct activation) and calcium imaging (direct activation) experiments when examining the effects of hemin on TRPV1 suggested that another variable may account for the direct activation seen in calcium imaging. Babes and colleagues previously demonstrated that PpIX is a strong photosensitizer causing a direct activation of both TRPA1 and TRPV1 when illuminated with the UVA-light (340/380 nm) used for calcium imaging [27]. We postulated that this property may also account for hemin. As is demonstrated in Figure 6A,C, co-application of hemin with the reducing agent DTT almost abolished the calcium influx induced by hemin (n = 379). Only 0.8% of capsaicin-sensitive cells were hemin-sensitive in presence of DTT. The mutant construct hTRPV1-3C was previously demonstrated to lack redox sensitivity, but to have an intact sensitivity to UVA-light [27,28]. Accordingly, the magnitude of hemin-induced calcium influx was significantly reduced on hTRPV1-3C (Figure 6B,C, n = 273). Furthermore, the percentage of hemin-sensitive cells dropped to 44% in hTRPV1-3C as compared to 58% for wildtype hTRPV1. When DTT was added to hemin in patch clamp experiments, we still observed a significant potentiation of proton-evoked currents (paired *t*-test, *p* < 0.01). The effect was not significantly reduced when compared to the effect induced by hemin alone (Figure 6D,E, n = 8, unpaired *t-*test, *p* = 0.17). DTT applied alone evoked a small but non-significant potentiation (Figure 6E, n = 8, paired *t-*test, *p* = 0.42). Hemin-induced potentiation of heat-evoked currents on hTRPV1 was not reduced by DTT (Figure 6F,G, unpaired *t-*test, *p* = 0.69). We also examined the effect of glutathione (GSH) applied in the pipette solution, e.g., another strong reducing agent which should prevent redox modification of hTRPV1. As is demonstrated in Figure 6H,I, 1 mM GSH did not prevent hemin-induced potentiation of proton-evoked inward currents (n = 9, paired *t-*test, *p* < 0.01). Thus, 1 mM GSH did not significantly alter hemin-induced sensitization of hTRPV1 (unpaired *t-*test, *p* = 0.19). The effect of hemin on proton-evoked currents was preserved on the hTRPV1-3C mutant (Figure 6J,K, n = 10, unpaired *t-*test, *p* = 0.17). These data suggest that hemin-induced activation of hTRPV1 observed in calcium imaging may be due to a photosensitizing effect of hemin applied together with UVA-light.

**Figure 7:**
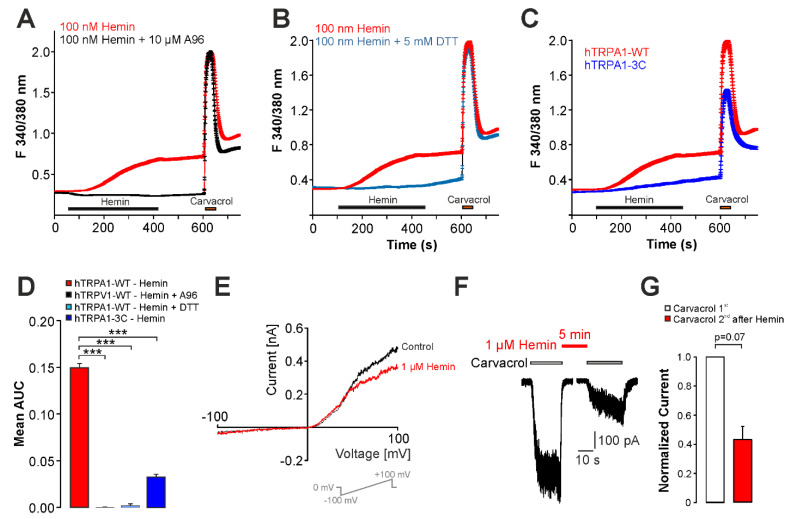
Hemin activates hTRPA1 expressed in HEK293t cells. (**A**) Hemin-induced (100 nM) calcium responses in hTRPA1-expressing cells with or without application of the TRPA1-inhibitor A967079 (10 µM). Hemin was applied for 300 s followed by washout and the TRPA1-agonist carvacrol (200 µM). (**B**) Mean calcium responses in hTRPA1-expressing cells treated with 100 nM hemin or hemin together with 5 mM DTT. (**C**) Mean calcium responses in cells expressing hTRPA1-WT or hTRPA1-3C treated with 100 nM hemin for 300 s. (**D**) Mean area under the curve (AUC) for the hemin-induced increase in fluorescence ratio for experiments described under (**A–C**). ANOVA F(3, 2583)=156.07, *p* < 0.001. (**E**) Representative whole-cell patch clamp recording on a HEK293t cell expressing hTRPA1. Membrane currents were evoked by a 500 ms long voltage-ramp from −100 to +100 mV. Note that hemin reduces the membrane current. (**F**) Whole-cell patch clamp recordings on hTRPA1-expressing cells challenged with two consecutive applications of carvacrol with 1 µM hemin applied for 5 min between both applications. (**G**) Mean normalized peak amplitudes of inward currents evoked by carvacrol (**F**). Current amplitudes were normalized to the amplitude of the first current. Cells were held at −60 mV in all patch clamp experiments. Data are shown as mean ± S.E.M. *** denotes *p* < 0.001.

If true, the combination of hemin and UVA-light should also activate TRPA1 which displays a higher redox sensitivity than TRPV1. Accordingly, the experiments performed on DRG neurons suggested that TRPA1 contributes to hemin-induced calcium influx. When we examined HEK293t cells expressing hTRPA1, we found that hemin (>10 nM) induced an increase in intracellular calcium which is completely inhibited by the selective TRPA1-inhibitor A967079 (Figure 7A,D, n = 1507 and 400, respectively). A967079 also reduced the number of hemin-sensitive carvacrol responding cells from 52% to 0%. Similar to the cells expressing hTRPV1, the co-application of hemin with DTT abolished hemin-induced calcium influx in hTRPA1-expressing cells (Figure 7B,D, n = 612). In presence of DTT, only 5% of carvacrol-sensitive cells responded to hemin. Furthermore, the redox-insensitive mutant hTRPA1-3C displayed a reduced hemin-induced calcium influx as compared to wildtype hTRPA1 (Figure 7C,D, n = 612, 14% hemin-sensitive cells). In patch clamp experiments however, 1 µM hemin failed to induce membrane currents in cells expressing hTRPA1 (Figure 7E, n = 6). Finally, hemin did not potentiate carvacrol-induced inward currents on hTRPA1 (Figure 7F,G, n = 12, paired *t-*test, *p* = 0.07).

## 3. Discussion

In this mechanistic in vitro study, we demonstrate that the porphyrin hemin is able to sensitize the capsaicin receptor TRPV1 in a PKC-dependent manner. Activation of TRPV1 was only observed in calcium imaging recordings, and our data strongly suggest that it is mediated by a UVA-light-induced accumulation of reactive oxygen species sufficient to activate TRPV1. This effect is thus most probably due to an in vitro artifact resulting from the yet poorly recognized property of hemin as a photosensitizer producing intracellular ROS when challenged with UVA-light.

To our knowledge, the potential effects of free hemin (or heme) on the excitability of nociceptive sensory neurons, or on specific ion channels involved in peripheral pain processing have never been reported on. Saying this, Chiabrando and co-workers recently pledged for an increased attention to the yet poorly examined role of hemin as a relevant player in nociception [29]. The authors mainly referred to recent findings showing that loss-of-function variants of the gene encoding for the heme export protein FLVCR1 (feline leukemia virus subgroup receptor 1) can cause hereditary sensory and autonomic neuropathies with a loss of pain perception [29,30,31]. A likely mechanism for this disorder is an accumulation of intracellular heme and a consecutive oxidative stress triggering neurotoxicity and thus sensory denervation [30]. On the other hand, Li and colleagues reported that intrathecal injection of hemin in naïve rats resulted in thermal and mechanical hyperalgesia [32]. Accordingly, hemin strongly upregulates HO-1, which was demonstrated to play an important role for pain-related behavior in several rodent models [32,33]. In the present study, we tested the hypothesis that hemin may modify the capsaicin receptor TRPV1, e.g., as a mechanism for direct activation of sensory neurons. Besides the fact that TRPV1 is polymodal, and is directly or indirectly gated by multiple substances [1], this hypothesis was based on recent studies demonstrating that TRPV1 is an important mechanism for the intensive pain associating sickling episodes in a mouse model for SCD [11]. On the other hand, TRPV1-expressing sensory neurons fulfill a protective function in the same mouse SCD model [4]. These findings thus implicate that TRPV1 is gated by endogenous substances being released in the course of SCD. Of note, the amount of free hemin seems to correlate with the severity of SCD [16,17]. Indeed, our data clearly demonstrate that hemin is able to sensitize or even activate TRPV1. However, our data need to be taken with caution as they—in part—result from an evident in vitro artifact with a limited physiological relevance. As TRPV1 displays a high permeability for calcium, FURA-2 based calcium imaging is very commonly utilized for experiments of function and pharmacology of TRPV1. Application of UVA-light is mandatory for this technique. We recently demonstrated that prolonged application of UVA-light results in a strong sensitization of TRPV1 [32]. This effect was, in part, due to the redox sensitivity of TRPV1 requiring C- and N-terminal cysteine residues. In the same study, as well as in previous studies, TRPA1, TRPV1, and TRPV2 were demonstrated to be activated by photosensitizing substances which produce a strong intracellular production of ROS when illuminated with UVA-light [27,34,35,36]. One such photosensitizer is PpIX, e.g., heme lacking the central Fe^2+^. Photosensitivity of hemin itself thus lies at hand, but in this regard, we were not alert or critical enough when encountering this study. We observed a very robust hemin-induced activation of both TRPV1 and TRPA1, both for recombinant channels in HEK293t cells and in mouse DRG neurons. Intriguingly, we also observed a TRPV1/TRPA1-independent calcium influx in both cell types when 10 µM hemin was applied. In naïve HEK293t cells, hemin even induced a small but robust release of calcium from internal stores. The data strongly suggest that either hemin itself or, more likely, hemin-induced accumulation of ROS is able to increase intracellular calcium by targeting several mechanisms. We did not further characterize these effects, but the inhibitory effect of ruthenium red observed in DRG neurons indicates that it is not due to an unspecific effect, but rather to yet unknown redox-sensitive mechanisms. Nevertheless, we question the physiological relevance of these findings. To our knowledge, there is no evident pathophysiological role of hemin associated with exposure to UVA-light. However, our data clearly show that in vitro assays utilizing UVA-light should be critically used when hemin or other porphyrins are explored. For example, previously published calcium imaging data showing hemin-induced calcium influx in cortical neurons may as well result from a photosensitizing property of hemin [18].

Importantly, we also found that hemin sensitizes TRPV1. This effect was independent of UVA-light, but diminished in PKC-insensitive mutant constructs of TRPV1 and by inhibition of PKC. Indeed, TRPV1 is known to be gated via a PKC-mediated phosphorylation [24]. In a recent study on a knock-in mouse carrying a PKC-insensitive TRPV1-mutant (TRPV1-S801A), the authors found that PKC sensitivity of TRPV1 can be important for inflammatory pain [23]. As hemin was described to activate PKC in previous studies as well [22], it is tempting to speculate that it might increase pain sensitivity due to this property. At this point, it should also be mentioned that Sahoo and colleagues demonstrated that hemin is a potent modulator of the potassium channel Kv1.4 such that is removes inactivation [11]. Incorporating this effect into neuronal excitability, it may reduce rather than increase action potential firing. As Kv1.4 and other hemin-sensitive potassium channels are expressed in sensory neurons, it could also be postulated that hemin is likely to induce analgesia. Of course, these cellular data not at all allow for such farfetched conclusions. Importantly, the concentrations of hemin used in our study seem to be of physiological relevance as plasma levels of free hemin up to 20 µM have been described [22]. Therefore, we believe that our data may encourage further investigations addressing the question if and how hemin can modify peripheral pain processing.

To conclude, our data identify hemin as an endogenous modulator of TRPV1. This property of hemin may be relevant for pain-related sequelae associated with increased tissue or plasma concentrations of hemin.

## 4. Materials and Methods

### 4.1. Cell Culture and cDNA

Using jetPEI (VWR, Darmstadt, Germany), HEK293t cells were transfected with different constructs of hTRPV1 or hTRPA1 as previously described [26]. Site directed mutagenesis on hTRPA1 and hTRPV1 cDNA was performed according to the instructions of the manufacturer (QuikChange lightning site-directed mutagenesis kit, Aglient, Waldbronn, Germany). All mutants were sequenced subsequently to exclude further channel mutations. Cells were cultured under standard conditions (5% CO_2_ at 37 °C) in Dulbecco’s modified Eagle medium nutrient mixture F12 (DMEM/F12 Gibco/Invitrogen, Darmstadt, Germany) supplemented with 10% fetal bovine serum (Biochrom, Berlin, Germany).

### 4.2. Chemicals

Capsaicin and A967079 were purchased from HelloBio (Bristol, UK), BCTC was purchased from Tocris (Wiesbaden-Nordenstadt, Germany). Ruthenium red, α1-antitrypsin, protoporphyrin-IX, hemin, iron(II) sulfide, sodium dithionite, DL-dithiothreitol, chelerythrine, reduced glutathione, and carvacrol were purchased from Sigma-Aldrich (Taufkirchen, Germany). Hemin was resuspended in 30 mM KOH to produce a 10 mM stock solution which was stored on ice. The dilutions needed for the measurements were freshly prepared before use and dissolved in external solution.

### 4.3. Whole-Cell Patch Clamp

An EPC10 USB HEKA amplifier (HEKA Elektronik, Lambrecht, Germany) was employed for whole-cell voltage clamp recordings. Signals were low passed at 1 kHz and sampled at 2 to 10 kHz. Patch pipettes were pulled from borosilicate glass tubes (TW150F-3; World Precision Instruments, Berlin, Germany), filled pipettes had a resistance of 2–4 MΩ. The external solution contained (in mM): NaCl 140, KCl 5, MgCl_2_ 2, EGTA 5, HEPES 10, and glucose 10 (pH 7.4 was adjusted with NaOH). Calcium was omitted in order to avoid desensitization of TRPV1. The pipette solution contained (in mM): KCl 140, MgCl_2_ 2, EGTA 5, and HEPES 10 (pH 7.4 was adjusted with KOH). A gravity-driven multi-barrel perfusion system was used for application of test solutions. For application of heated solution, the Patchmaster software (HEKA Elektronik, Lambrecht, Germany) was used to control an application system allowing precise and time-controlled heating of solution at the tip of the application capillary as described previously [26]. Data acquisition and analyses were performed with Patchmaster/Fitmaster (HEKA Elektronik, Lambrecht, Germany) and Origin 8.5.1 (Origin Lab, Northampton, MA, USA) software.

### 4.4. Calcium Imaging

Cells were stained with Fura-2 AM (3 µM) and 0.01% pluronic F-127 (both from Biotium Inc., Fremont, CA, USA) for about 1 h. Coverslips were mounted on an inverse microscope. The extracellular solution contained (in mM): NaCl 145, KCl 5, CaCl_2_ 1.25, MgCl_2_ 1, Glucose 10, Hepes 10). For calcium-free experiments, CaCl2 was replaced by 5 mM EGTA. Solutions were applied using a gravity-driven superfusion system. Fura-2 was excited using a microscope light source and an LEP filter wheel (Ludl electronic producs Ltd., Hathorne, CA, USA) to switch between 340 and 380 nm. Images were exposed for 20 and 10 ms, respectively, and acquired at a rate of 1 Hz with a CCD camera (Cool SNAP EZ, Photometrics). Data were recorded using VisiView 2.1.1 software (all from Visitron Systems GmbH, Puchheim, Germany). Background fluorescence was subtracted before calculation of ratios. A 60 mM potassium chloride stimulus was applied as a control at the end of each experiment for DRG neurons and 5 µM ionomycin was used in HEK293t cells. Cells which did not respond to potassium/ionomycin, or showed no functional expression of TRPV1 or TRPA1 in experiments on HEK293t cells, were excluded from the analysis. Averaged results are reported as means (± S.E.M.).

### 4.5. Dorsal Root Ganglion Neuron Culture

DRG neurons from C57Bl/6 wildtype, TRPA1, TRPV1, and TRPV1/TRPA1 knockout mice was performed as described previously [26]. Briefly, mice were deeply anaesthetized by isoflurane inhalation, sacrificed by decapitation, and DRGs from all levels were excised and transferred to Dulbecco’s modified Eagle’s medium (DMEM). Following treatment with DMEM containing 1 mg/ml collagenase and 0.5 mg/mL protease for 45 min (both from Sigma-Aldrich, Taufkirchen, Germany), ganglia were dissociated using a fire-polished, silicone-coated Pasteur pipette. Isolated cells were transferred onto poly-L-lysine-coated (0.1 mg/mL, Sigma Aldrich) coverslips and cultured in TNB 100 medium supplemented with TNB 100 lipid protein complex, penicillin/streptomycin (100 U/mL) (all from Biochrom, Berlin, Germany), and mouse NGF (100 ng/mL, Almone Laboratories, Tel Aviv, Israel). Cells were used for experiments within 24 h after plating. TRPA1-/- and TRPV1-/-TRPA1-/- adult mice of both genders were donated by Professor Dr. Peter Reeh (Institute of Physiology and Pathophysiology, University of Erlangen-Nuremberg, Erlangen, Germany). All procedures of this study were approved by the animal protection authorities (local district government, Hannover, Germany).

### 4.6. Statistical Analysis

Statistical analysis was performed using Origin 8.5.1 (Origin Lab, Northampton, MA, USA) or Statistica 7.1 (Stat soft Inc., Tulsa, OK, USA). All data are presented as mean ± S.E.M. Patch clamp data were analyzed using paired or unpaired students *t-*tests. Cells used for analyses were collected from at least two experimental days. PCR experiments were analyzed by Kruskal Wallis test followed by Dunn’s multiple comparison test. For analysis of calcium imaging more than two groups were compared using ANOVA, followed by Tukey HSD post hoc test. If not otherwise noted, significance was assumed for *p* < 0.05. In all figure legends. * denotes *p* < 0.05, ** denotes *p* < 0.01, and *** denotes *p* < 0.001.

## Data Availability

The data presented in this study are contained within the article. Original data for electrophysiology are available on request from the corresponding author.
